# IgA nephropathy associated with thalassemia: a case report

**DOI:** 10.1186/s12882-020-01844-3

**Published:** 2020-05-14

**Authors:** Jun Ni, Caifeng Zhu, Xiaoqiu Ni, Jiazhen Yin

**Affiliations:** 1 The Department of Nephrology (Key laboratory of management of kidney disease in Zhejiang province), Hangzhou Hospital of Traditional Chinese Medicine, Hangzhou, Zhejiang P. R. China; 2 Zhejiang Chinese Medical University, Hangzhou, Zhejiang P. R. China

**Keywords:** α-Thalassemia, IgA nephropathy, Renal complications

## Abstract

**Background:**

Thalassemia is a group of hereditary diseases characterized by a common recessive monogenic hematological disorder, presenting a significant public health concern in the developing countries. Recent studies have identified the renal effects of thalassemia syndrome. Chronic hypoxia, long-term anemia, iron overload, and iron chelators are the major causes of renal tubular dysfunction and glomerular filtration abnormalities, while glomerulonephritis is not considered a major cause of abnormal urinalysis.

**Case presentation:**

We report a case of a 38-year-old female patient with immunoglobulin A (IgA) nephropathy accompanied by anemia who was misdiagnosed initially, but was diagnosed with alpha-thalassemia after gene tests. We administered a combination of oral prednisolone, leflunomide, and angiotensin receptor blockers as well as folic acid and mecobalamin. During the follow-up, her proteinuria was significantly reduced, and her anemia was improved.

**Conclusions:**

The possibility of occurrence of thalassemia should be considered in IgA nephropathy complicated with refractory anemia, especially in high-incidence areas of the disease.

## Background

Thalassemia refers to a group of hereditary hemolytic anemias, wherein mutations or deletions of the globin gene lead to various degrees of inhibition in α or β globin synthesis. The clinical manifestations are correlated with the severity of the disease. The manifestations primarily include anemia, jaundice, and hepatosplenomegaly. Population mobility and migration have allowed the disease to become increasingly common. The prevalence of α-thalassemia and β-thalassemia in China was 7.88 and 2.21%, respectively and the diseases were mainly distributed in the southern China [1]. Recent studies have identified the proximal tubular damage and glomerular filtration abnormalities in thalassemia. However, to the best of our knowledge, immunoglobulin A (IgA) nephropathy (IgAN) has rarely been reported in patients with thalassemia. The present report describes the first case of IgAN that is associated with α-thalassemia.

## Case presentation

A 38-year-old female was admitted to our center with a complaint of abnormal urine tests. She was diagnosed with chronic nephritis syndrome 10 years ago, but did not receive appropriate medical care during this period. The patient also complained of menorrhagia with unexplained anemia. Physical examination revealed no positive signs such as bilateral lower limb edema or hepatosplenomegaly. Urinalysis revealed proteinuria (2+), blood (3+), and more than 75% the dysmorphic red blood corpuscles. The protein content was 0.71 g in 24-h urine analysis. The urinary N-acetyl-β-D-glucosaminidase was 11.14 U/g·Cr, and urinary α1 microglobulin was 12.6 mg/L. In additional renal function tests, creatinine clearance rate was 96.1 ml/min, glomerular filtration rate (GFR) was 93.1 ml/min; and serum creatinine was 71 μmol/L. The albumin content was 34.5 g/L, and the results of thyroid function tests were within the normal ranges. Tests for anti-neutrophil cytoplasmic antibodies, anti-nuclear antibodies and anti-double-stranded DNA antibodies were negative. No viral markers of hepatitis were detectable, and all the antibodies were negative except the surface antibodies. Levels of serum IgA and complement component C3 were also normal. Ferritin level was 44.1 ng/mL. Complete blood tests revealed microcytic hypochromic anemia. Hemoglobin (Hb) was 84 g/L, mean corpuscular volume was 69 fL, and mean corpuscular Hb was 22 pg. Ultrasonography results of kidney were normal. Results of renal biopsy showed that 15 glomeruli were detected by light microscopy and one of them exhibited fibrocellular crescents. Renal tubules displayed localized atrophy, and inflammatory cell infiltration as well as fibrosis in the interstitium. Arteries and arterioles were unremarkable. Immunofluorescent analysis revealed diffuse IgA (3+) and C3 (2+) staining were present in several mesangial areas. Based on these results, the patient was diagnosed with IgAN accompanied by iron deficiency anemia. We treated the patient with a combination of oral prednisolone, leflunomide, and angiotensin receptor blockers (ARBs). In addition, polysaccharide-iron complex was also administered. A complete remission of proteinuria was observed, and hematuria was also relieved. However, Hb was constantly under the normal reference range, which could not be explained by the iron deficiency anemia. In 2016, the patient was diagnosed with α-thalassemia based on the results of our genetic tests showing the deletion of --SEA gene. Serum HbF and HbA2 were elevated to 2.47 and 3.04%, respectively. During the 3-year follow-up, a significant improvement in anemia was observed following the treatment consisting of a small dose of ARB with folic acid and mecobalamin administration. Laboratory tests in March 2019 showed that Hb levels were elevated to 157 g/L. Urine occult blood was 2+, 24-h proteinuria was reduced to 0.08 g, and serum creatinine and albumin were normal.

## Discussion and conclusions

Thalassemia is a common inherited disease of hemoglobin synthesis worldwide. Patients with thalassemia have a wide spectrum of clinical phenotypes depending on their respective genotype characteristics. Some patients with thalassemia may not have any clinical symptoms, leading to misdiagnosis. Patients with thalassemia major, the most severe type, usually require regular blood transfusions for survival and some patients even die in utero or shortly after birth. The mortality of thalassemia is considerable, particularly among children [2].

Although clinical manifestations of thalassemia can be accompanied by multisystem complications including cardiopulmonary dysfunction and endocrine organ disease, kidney involvement has received less attention. Previous clinical studies have identified thalassemia-associated proximal tubular dysfunction and abnormalities in GFR [3], which have been confirmed in animal models as well [4]. Ahmadzadeh et al. [5] reported that 140 Iranian children with β-thalassemia had elevated levels of urinary N-acetyl-β-D-glucosaminidase and ferritin, suggesting tubular dysfunction. Moreover, proteinuria and microscopic hematuria were also observed in children with β-thalassemia, suggesting the probable association of glomerular disease with thalassemia.

The pathogenesis of kidney dysfunction among patients with thalassemia may involve chronic hypoxia that causes proximal tubular cells dysfunction and interstitial fibrosis with consequent renal damage. Anemia induces renal hemodynamic changes in the presence of other risk factors [6, 7]. Nickavar [8] reported that almost 20% of the patients with β-thalassemia minor had relatively low urine osmolality, suggesting tubular dysfunction. In the present case, the patient also showed elevated urinary N-acetyl-β-D-glucosaminidase and α1 microglobulin levels. In patients who receive regular transfusion, the destruction of glomerular filtration barrier is also attributed to iron overload secondary to regular blood transfusion, resulting in an increase of oxidative stress and direct cytotoxicity, which in turn leads to a decline in the ability of the kidneys to clear immune complexes (ICs) [9]. The use of specific iron chelators may also lead to kidney damage. Some clinical reports have found that the occurrence of proteinuria in thalassemia is correlated with iron toxicity [10]. Microscopic hematuria is often observed in thalassemia patients, which may be related to tubular dysfunction induced by hyperuricosuria, hypercalciuria, iron overload, or deferoxamine. Glomerulonephritis is not considered as a major cause of microscopic hematuria.

Thalassemia associated with IgAN is relatively rare. In 1994, Harada [11] reported a case of a beta-thalassemia patient with microscopic hematuria, which was later diagnosed with IgAN. Jung Hyun Kang et al. [12] reported a case of a beta-thalassemia minor patient who did not receive any specific treatment for anemia. During the follow-up, the patient exhibited overt microscopic hematuria and proteinuria with elevated serum IgA levels and was diagnosed with IgAN after renal biopsy. In 2016, Mrabet [13] also reported a case of a beta-thalassemia minor patient associated with IgAN who showed persistent microscopic hematuria and new-onset overt proteinuria.

ICs are considered important pathogenic factors of IgAN. Factors affecting the deposition of ICs in glomeruli are mainly the one associated with the ability of the body to clear ICs. The elimination of ICs in human circulation is mostly accomplished by erythrocytes through immune adhesion and phagocytosis. When erythrocyte immune function is impaired or the complement-dependent clearance mechanism is affected by immune deficiency, the activity of complement receptor type 1 on erythrocyte membrane is decreased. Its ability of recognizing, adhering to, and clearing ICs is also decreased [14]. Therefore, ICs in the circulation are abnormally deposited in the mesangial area while being filtered by the glomerulus. This results in mesangial cell proliferative response, which constitutes the histopathological basis of IgAN. Thus, in patients with thalassemia, impaired elimination of circulating ICs possibly leads to IgAN.

The present report is the first one describing IgAN associated with α-thalassemia. The occurrence of IgAN in this case may be related to the impaired clearance of circulating ICs caused by thalassemia. However, the possibility of a concomitant existence of these two diseases could not be excluded, as both are common diseases in China. The patient has been followed up for more than 7 years and her condition is relatively stable under the current treatment. While treating with patients having urinary abnormalities combined with anemia, especially in cases wherein the renal function is insufficient, thalassemia is more likely to be misdiagnosed or missed, even in high incidence areas of the disease.

## Data Availability

Data regarding this study were obtained from clinical charts stored in the physician office records of Hangzhou Hospital of Traditional Chinese Medicine, therefore, cannot be shared. Any reasonable request to access the data must be approved before the data can be released.

## Abbreviations

IgAN: Immunoglobulin A nephropathy
GFR: Glomerular filtration rate
Hb: Hemoglobin
ARBs: Angiotensin receptor blockers
ICs: Immune complexes
